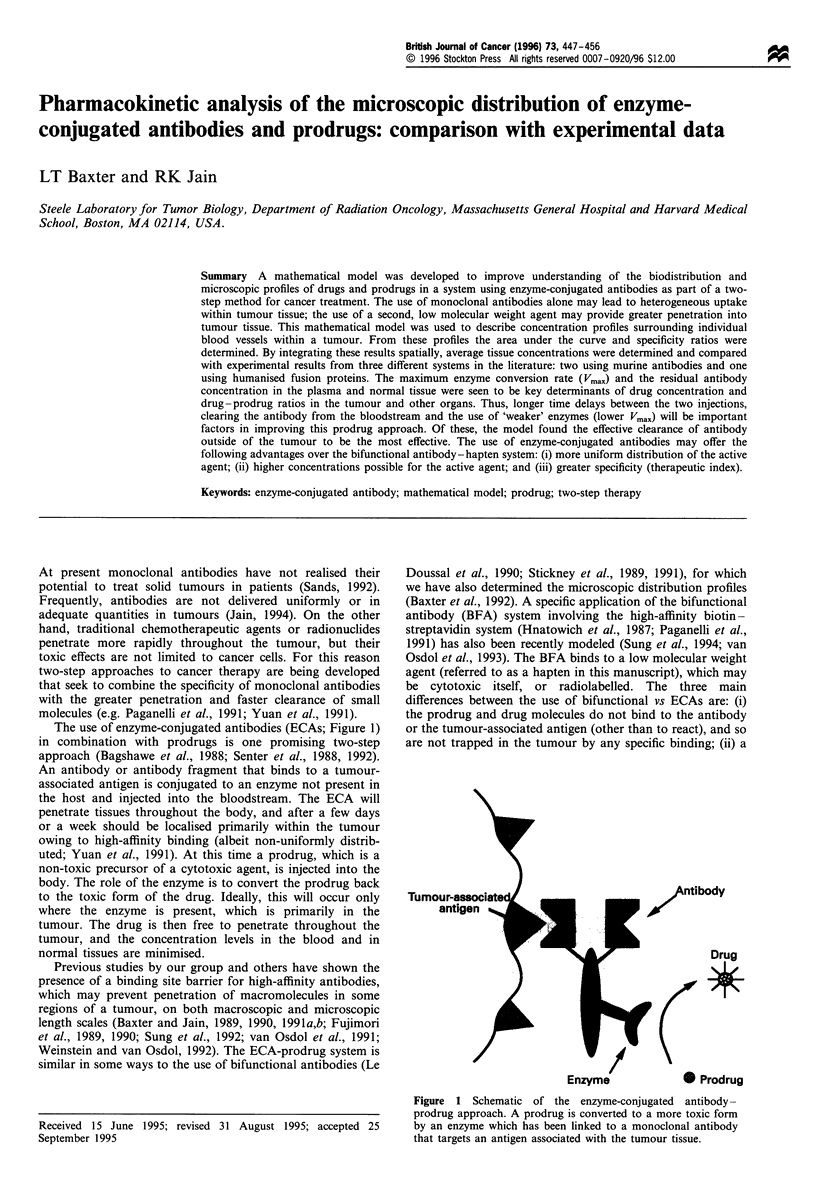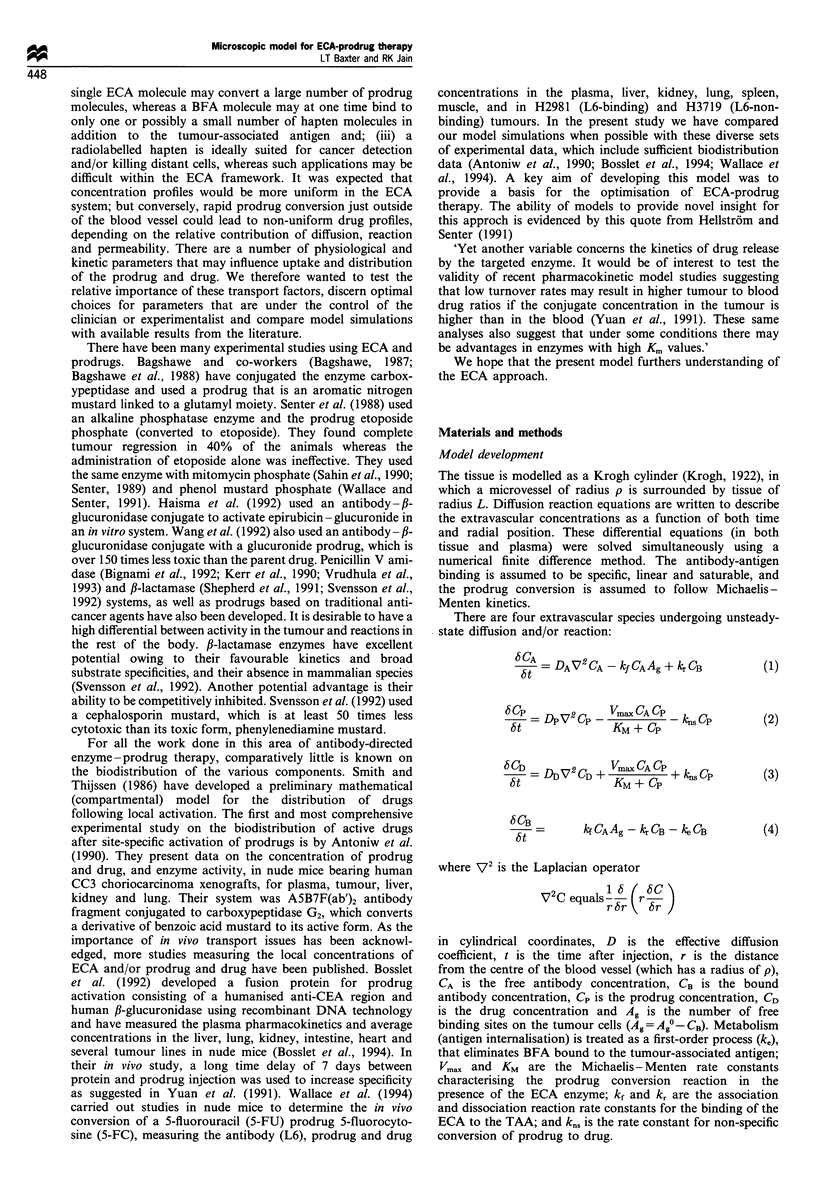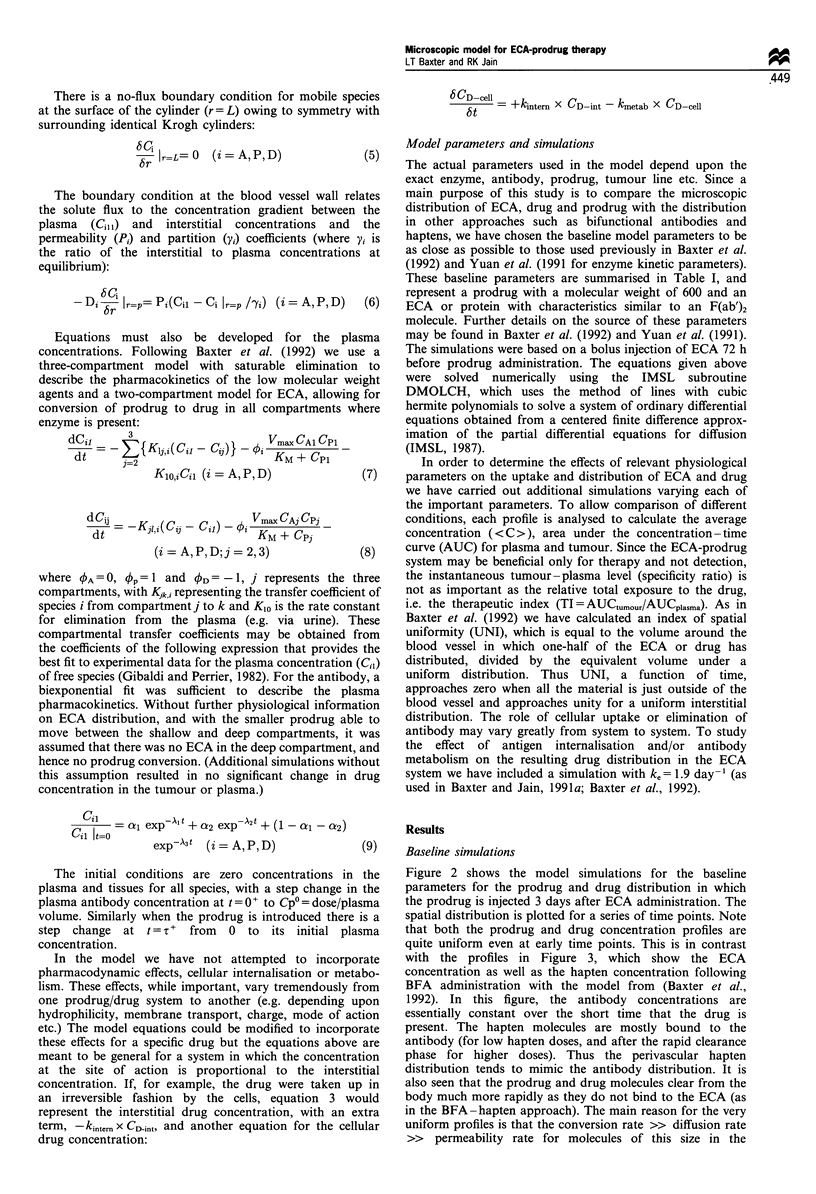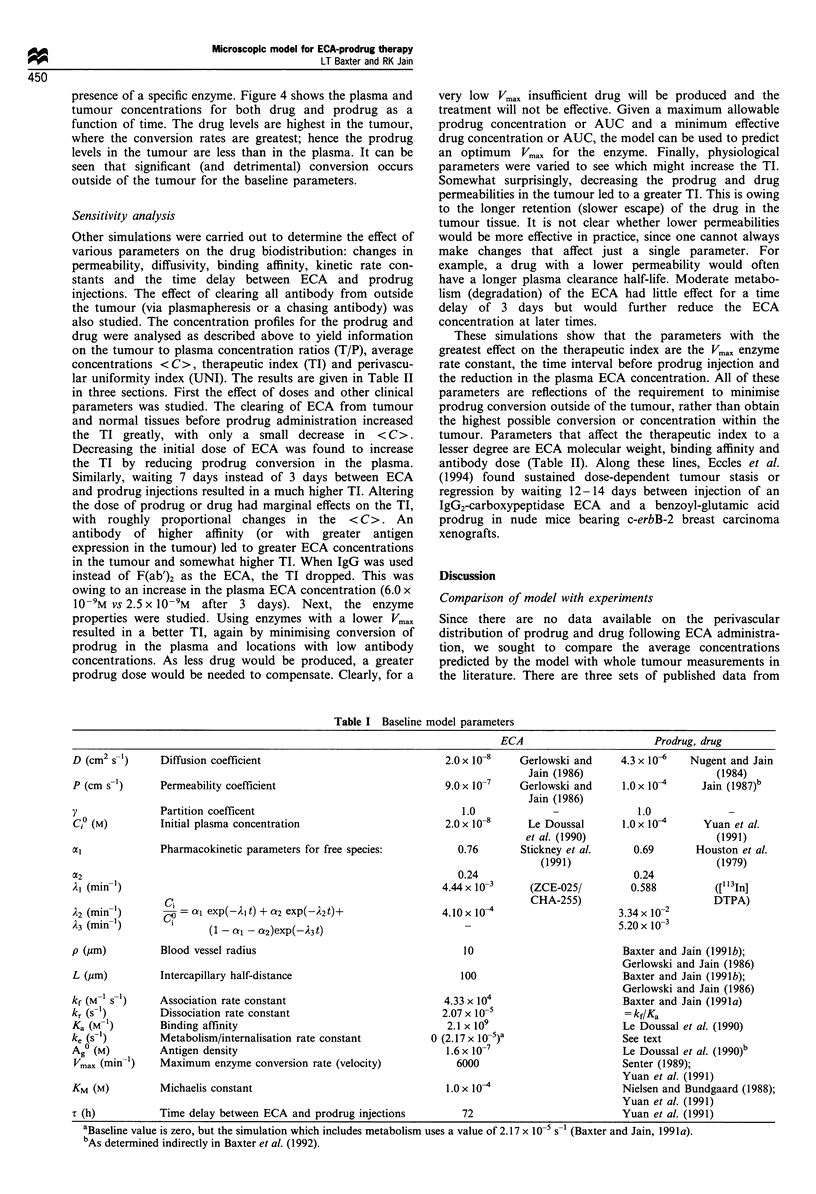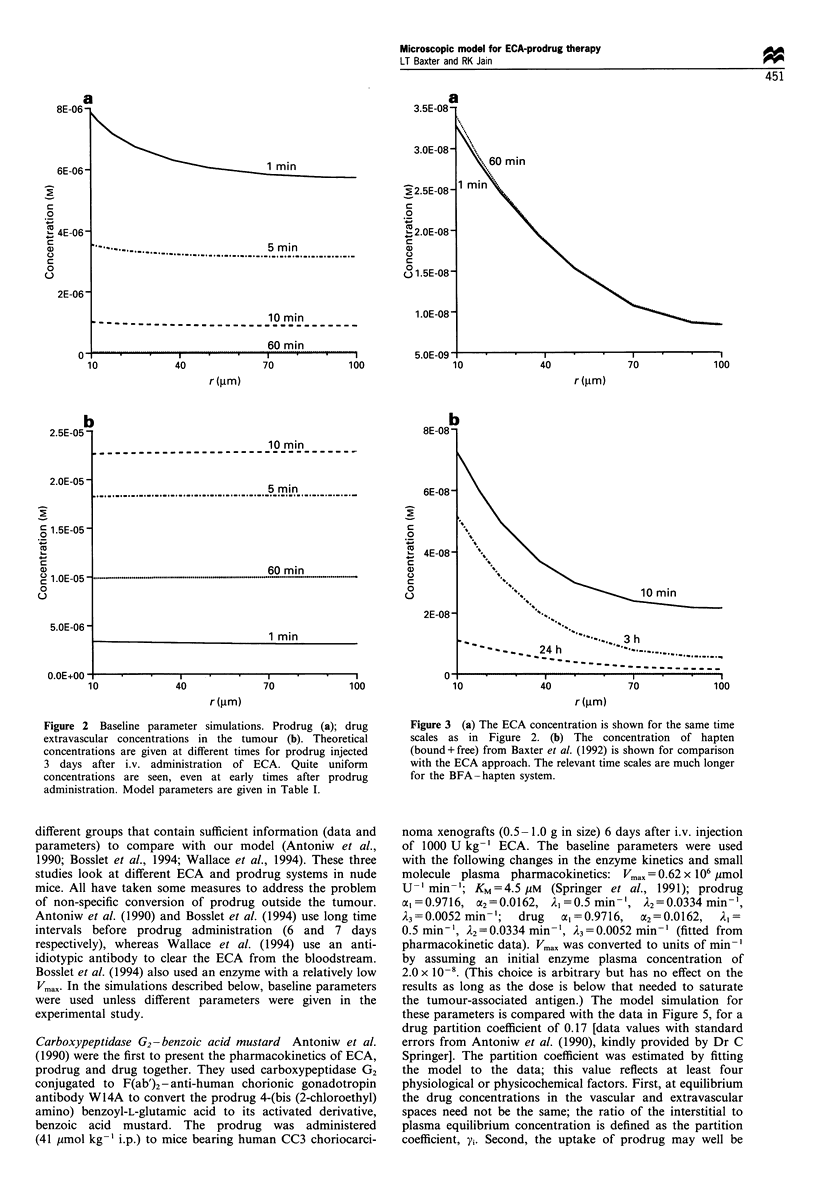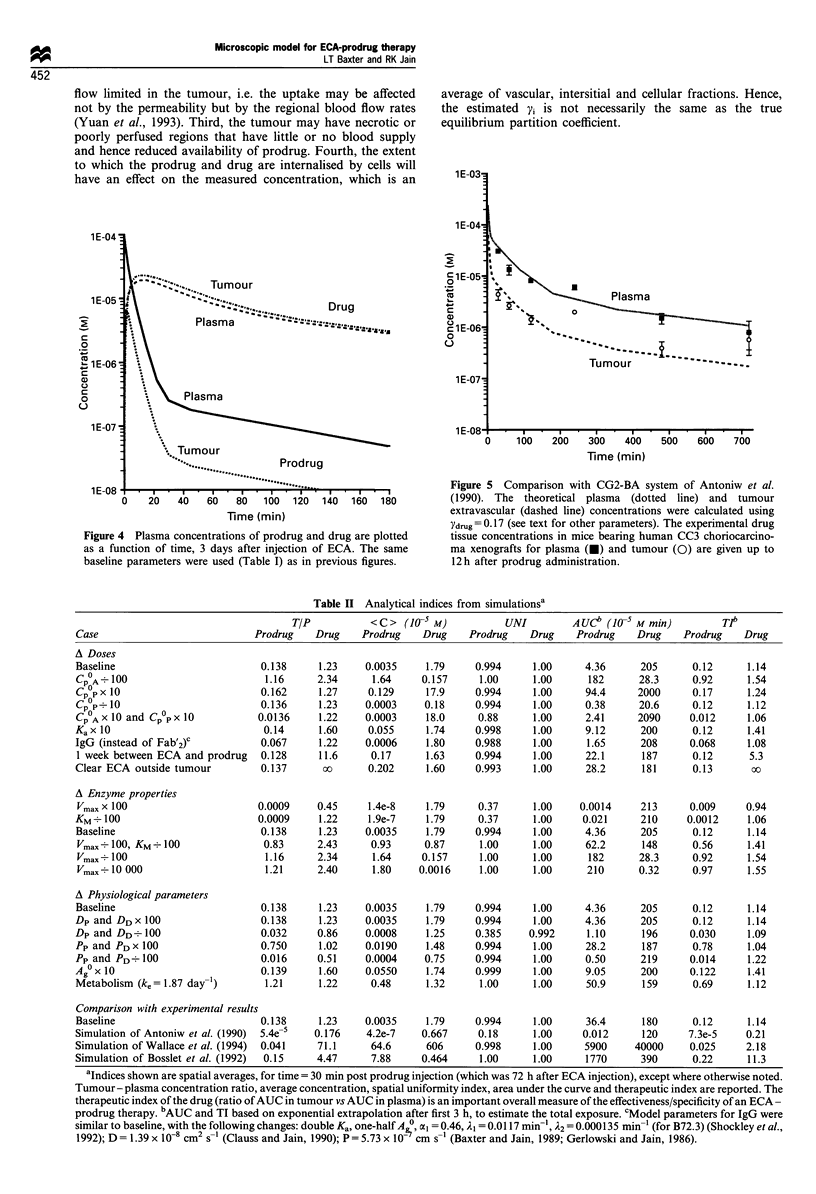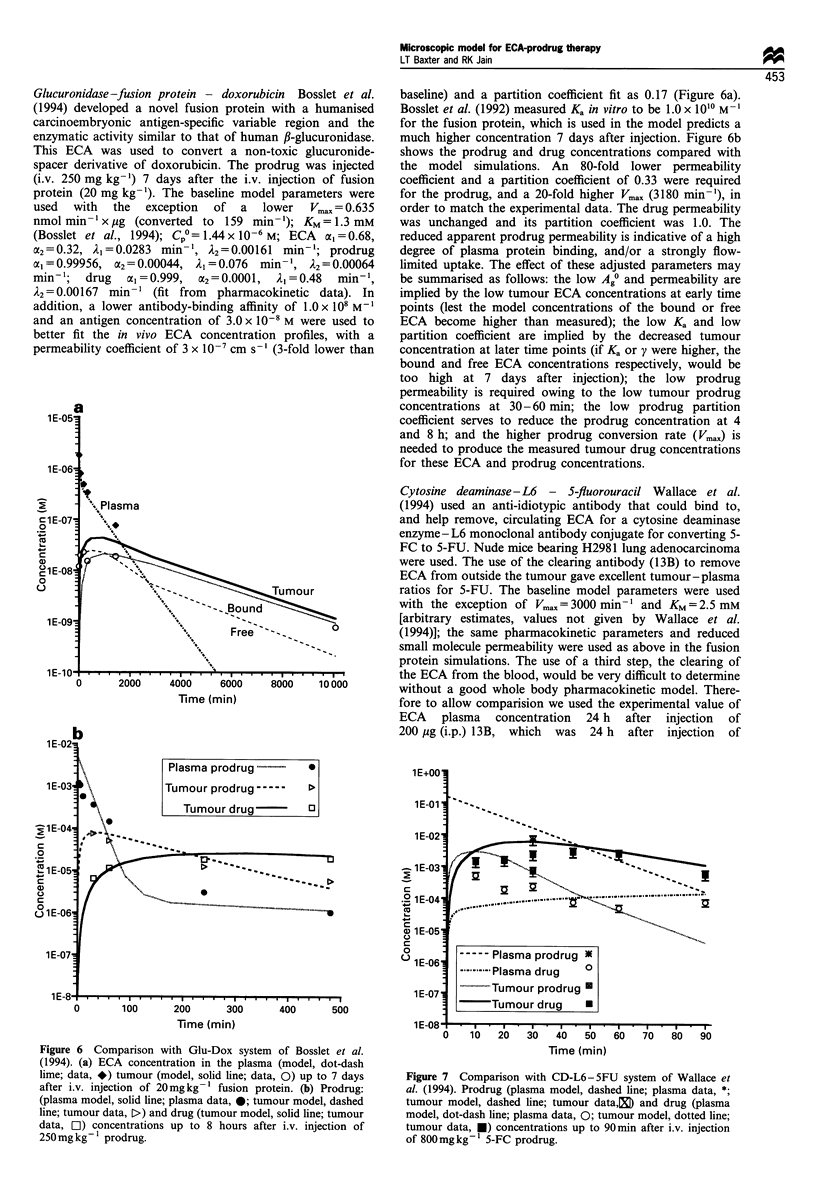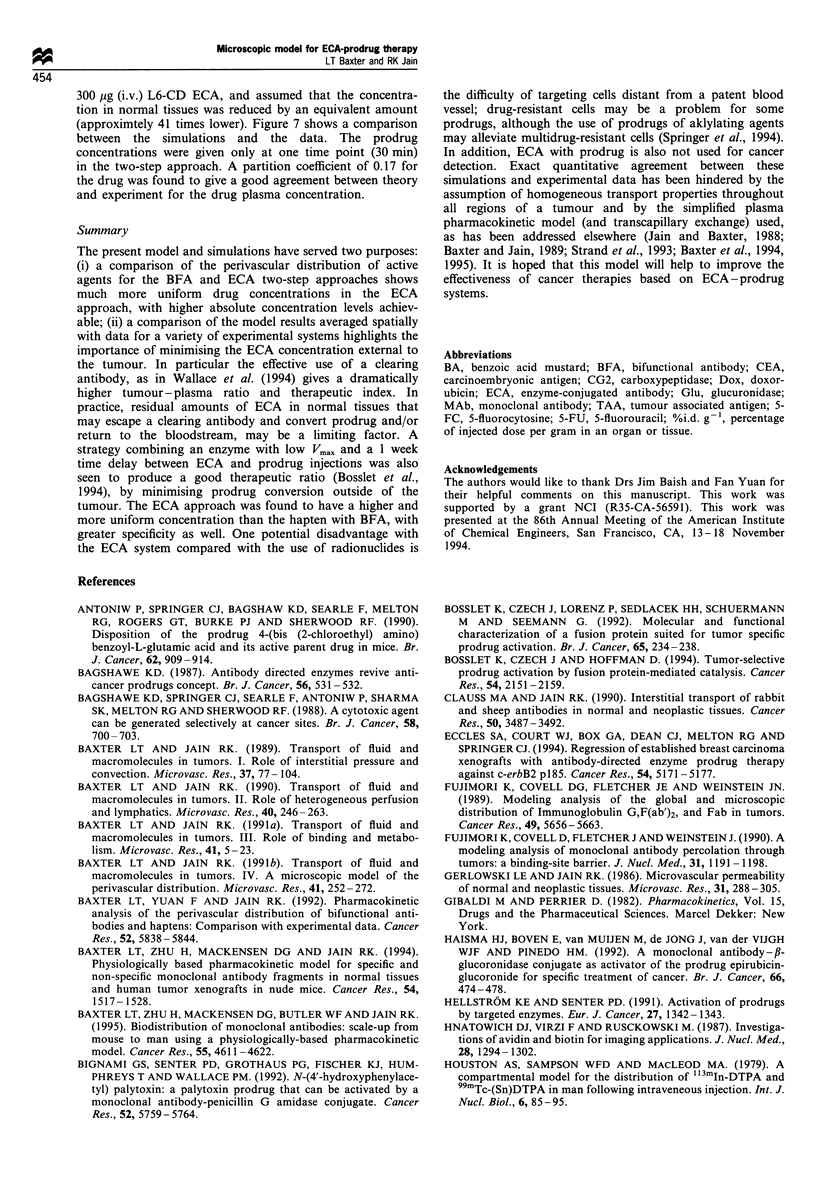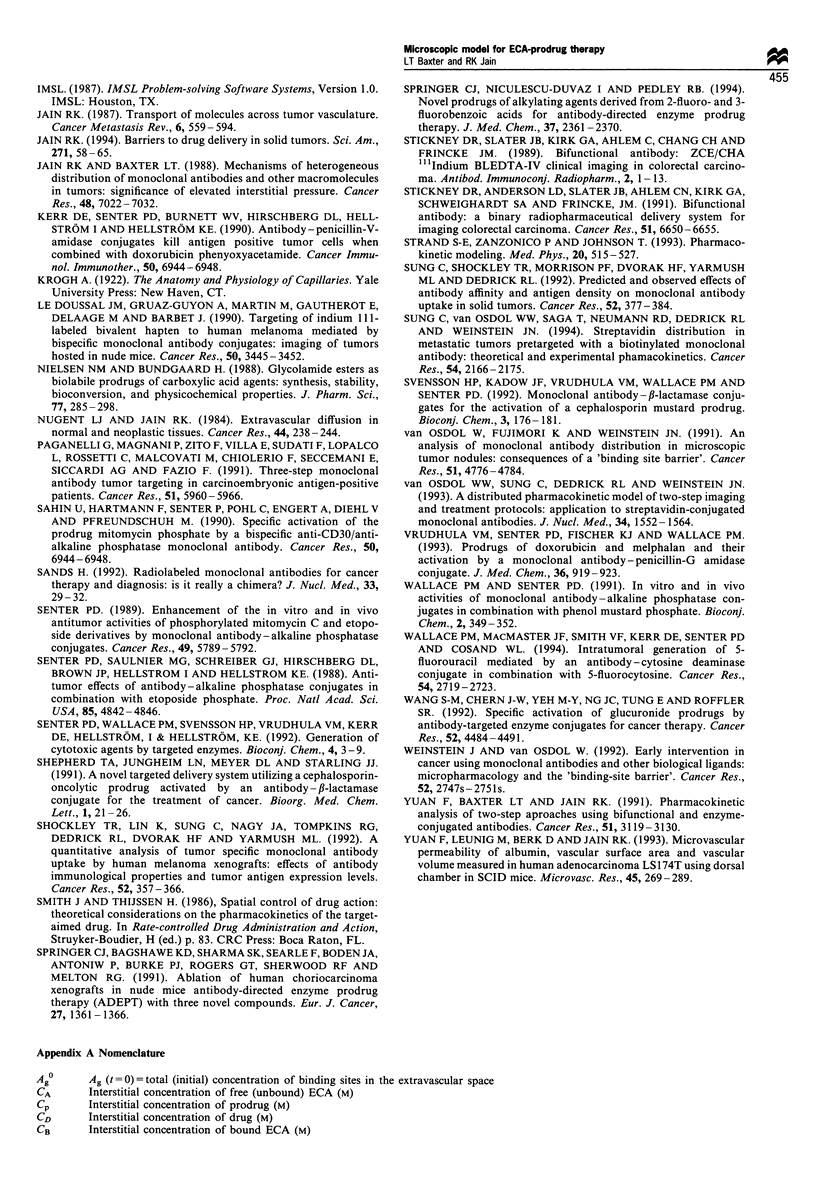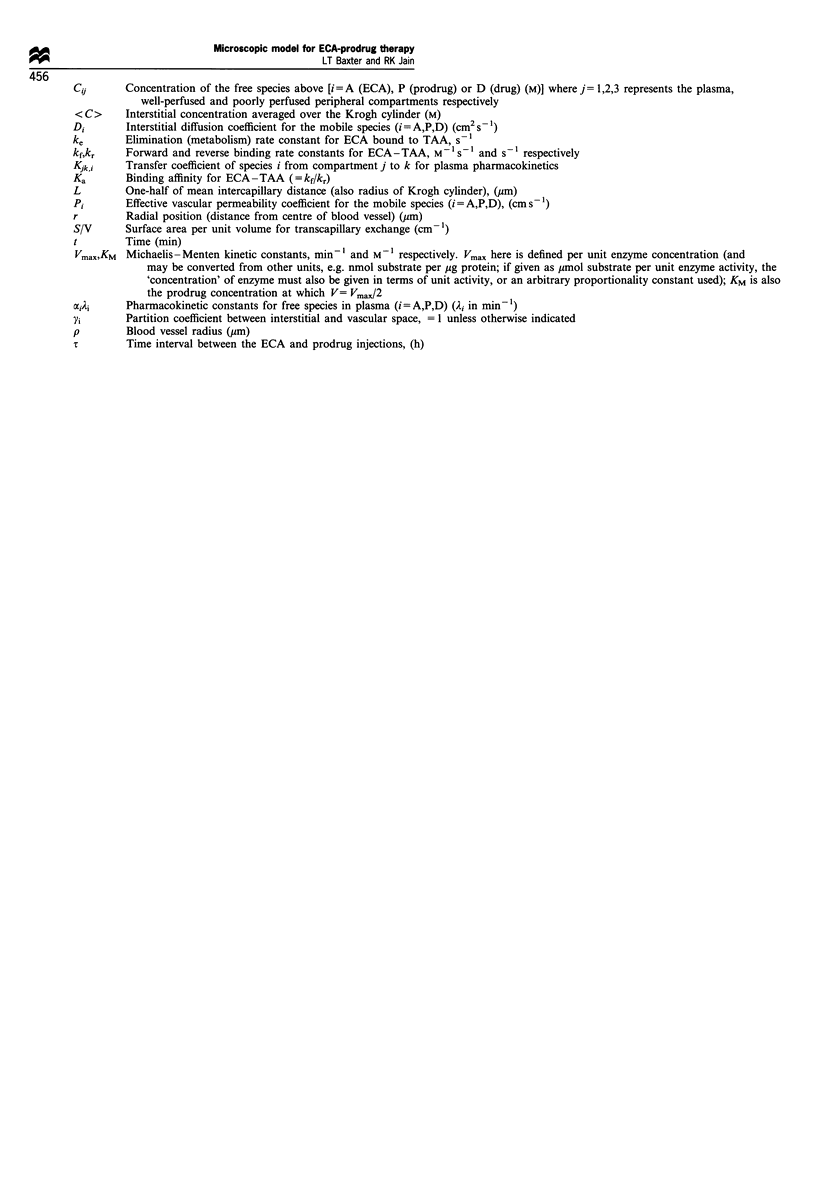# Pharmacokinetic analysis of the microscopic distribution of enzyme-conjugated antibodies and prodrugs: comparison with experimental data.

**DOI:** 10.1038/bjc.1996.80

**Published:** 1996-02

**Authors:** L. T. Baxter, R. K. Jain

**Affiliations:** Department of Radiation Oncology, Massachusetts General Hospital, Boston, USA.

## Abstract

A mathematical model was developed to improve understanding of the biodistribution and microscopic profiles of drugs and prodrugs in a system using enzyme-conjugated antibodies as part of a two-step method for cancer treatment. The use of monoclonal antibodies alone may lead to heterogeneous uptake within the tumour tissue; the use of a second, low molecular weight agent may provide greater penetration into tumour tissue. This mathematical model was used to describe concentration profiles surrounding individual blood vessels within a tumour. From these profiles the area under the curve and specificity ratios were determined. By integrating these results spatially, average tissue concentrations were determined and compared with experimental results from three different systems in the literature; two using murine antibodies and one using humanised fusion proteins. The maximum enzyme conversion rate (Vmax) and the residual antibody concentration in the plasma and normal tissue were seen to be key determinants of drug concentration and drug-prodrug ratios in the tumour and other organs. Thus, longer time delays between the two injections, clearing the antibody from the blood stream and the use of 'weaker' enzymes (lower Vmax) will be important factors in improving this prodrug approach. Of these, the model found the effective clearance of the antibody outside of the tumour to be the most effective. The use of enzyme-conjugated antibodies may offer the following advantages over the bifunctional antibody-hapten system: (i) more uniform distribution of the active agent; (ii) higher concentrations possible for the active agent; and (iii) greater specificity (therapeutic index).


British Journal of Cancer (1996) 73, 447-456
© 1996 Stockton Press All rights reserved 0007-0920/96 $12.00

### Pharmacokinetic analysis of the microscopic distribution of enzyme-conjugated antibodies and prodrugs: comparison with experimental data

LT Baxter and RK Jain

*Steele Laboratory for Tumor Biology, Department of Radiation Oncology, Massachusetts General Hospital and Harvard Medical School, Boston, MA 02114, USA.*

**Summary** A mathematical model was developed to improve understanding of the biodistribution and microscopic profiles of drugs and prodrugs in a system using enzyme-conjugated antibodies as part of a two-step method for cancer treatment. The use of monoclonal antibodies alone may lead to heterogeneous uptake within tumour tissue; the use of a second, low molecular weight agent may provide greater penetration into tumour tissue. This mathematical model was used to describe concentration profiles surrounding individual blood vessels within a tumour. From these profiles the area under the curve and specificity ratios were determined. By integrating these results spatially, average tissue concentrations were determined and compared with experimental results from three different systems in the literature: two using murine antibodies and one using humanised fusion proteins. The maximum enzyme conversion rate ($V_{max}$) and the residual antibody concentration in the plasma and normal tissue were seen to be key determinants of drug concentration and drug-prodrug ratios in the tumour and other organs. Thus, longer time delays between the two injections, clearing the antibody from the bloodstream and the use of 'weaker' enzymes (lower $V_{max}$) will be important factors in improving this prodrug approach. Of these, the model found the effective clearance of antibody outside of the tumour to be the most effective. The use of enzyme-conjugated antibodies may offer the following advantages over the bifunctional antibody-hapten system: (i) more uniform distribution of the active agent; (ii) higher concentrations possible for the active agent; and (iii) greater specificity (therapeutic index).

**Keywords:** enzyme-conjugated antibody; mathematical model; prodrug; two-step therapy

At present monoclonal antibodies have not realised their potential to treat solid tumours in patients (Sands, 1992). Frequently, antibodies are not delivered uniformly or in adequate quantities in tumours (Jain, 1994). On the other hand, traditional chemotherapeutic agents or radionuclides penetrate more rapidly throughout the tumour, but their toxic effects are not limited to cancer cells. For this reason two-step approaches to cancer therapy are being developed that seek to combine the specificity of monoclonal antibodies with the greater penetration and faster clearance of small molecules (e.g. Paganelli *et al.*, 1991; Yuan *et al.*, 1991).

The use of enzyme-conjugated antibodies (ECAs; Figure 1) in combination with prodrugs is one promising two-step approach (Bagshawe *et al.*, 1988; Senter *et al.*, 1988, 1992). An antibody or antibody fragment that binds to a tumour-associated antigen is conjugated to an enzyme not present in the host and injected into the bloodstream. The ECA will penetrate tissues throughout the body, and after a few days or a week should be localised primarily within the tumour owing to high-affinity binding (albeit non-uniformly distributed; Yuan *et al.*, 1991). At this time a prodrug, which is a non-toxic precursor of a cytotoxic agent, is injected into the body. The role of the enzyme is to convert the prodrug back to the toxic form of the drug. Ideally, this will occur only where the enzyme is present, which is primarily in the tumour. The drug is then free to penetrate throughout the tumour, and the concentration levels in the blood and in normal tissues are minimised.

Previous studies by our group and others have shown the presence of a binding site barrier for high-affinity antibodies, which may prevent penetration of macromolecules in some regions of a tumour, on both macroscopic and microscopic length scales (Baxter and Jain, 1989, 1990, 1991*a,b*; Fujimori *et al.*, 1989, 1990; Sung *et al.*, 1992; van Osdol *et al.*, 1991; Weinstein and van Osdol, 1992). The ECA-prodrug system is similar in some ways to the use of bifunctional antibodies (Le Doussal *et al.*, 1990; Stickney *et al.*, 1989, 1991), for which we have also determined the microscopic distribution profiles (Baxter *et al.*, 1992). A specific application of the bifunctional antibody (BFA) system involving the high-affinity biotin-streptavidin system (Hnatowich *et al.*, 1987; Paganelli *et al.*, 1991) has also been recently modeled (Sung *et al.*, 1994; van Osdol *et al.*, 1993). The BFA binds to a low molecular weight agent (referred to as a hapten in this manuscript), which may be cytotoxic itself, or radiolabelled. The three main differences between the use of bifunctional *vs* ECAs are: (i) the prodrug and drug molecules do not bind to the antibody or the tumour-associated antigen (other than to react), and so are not trapped in the tumour by any specific binding; (ii) a

**Figure 1** Schematic of the enzyme-conjugated antibody-prodrug approach. A prodrug is converted to a more toxic form by an enzyme which has been linked to a monoclonal antibody that targets an antigen associated with the tumour tissue.

Received 15 June 1995; revised 31 August 1995; accepted 25 September 1995

single ECA molecule may convert a large number of prodrug molecules, whereas a BFA molecule may at one time bind to only one or possibly a small number of hapten molecules in addition to the tumour-associated antigen and; (iii) a radiolabelled hapten is ideally suited for cancer detection and/or killing distant cells, whereas such applications may be difficult within the ECA framework. It was expected that concentration profiles would be more uniform in the ECA system; but conversely, rapid prodrug conversion just outside of the blood vessel could lead to non-uniform drug profiles, depending on the relative contribution of diffusion, reaction and permeability. There are a number of physiological and kinetic parameters that may influence uptake and distribution of the prodrug and drug. We therefore wanted to test the relative importance of these transport factors, discern optimal choices for parameters that are under the control of the clinician or experimentalist and compare model simulations with available results from the literature.

There have been many experimental studies using ECA and prodrugs. Bagshawe and co-workers (Bagshawe, 1987; Bagshawe *et al.,* 1988) have conjugated the enzyme carboxypeptidase and used a prodrug that is an aromatic nitrogen mustard linked to a glutamyl moiety. Senter *et al.* (1988) used an alkaline phosphatase enzyme and the prodrug etoposide phosphate (converted to etoposide). They found complete tumour regression in 40% of the animals whereas the administration of etoposide alone was ineffective. They used the same enzyme with mitomycin phosphate (Sahin *et al.*, 1990; Senter, 1989) and phenol mustard phosphate (Wallace and Senter, 1991). Haisma *et al.* (1992) used an antibody-$\beta$-glucuronidase conjugate to activate epirubicin-glucuronide in an *in vitro* system. Wang *et al.* (1992) also used an antibody-$\beta$-glucuronidase conjugate with a glucuronide prodrug, which is over 150 times less toxic than the parent drug. Penicillin V amidase (Bignami *et al.*, 1992; Kerr *et al.*, 1990; Vrudhula *et al.*, 1993) and $\beta$-lactamase (Shepherd *et al.*, 1991; Svensson *et al.*, 1992) systems, as well as prodrugs based on traditional anticancer agents have also been developed. It is desirable to have a high differential between activity in the tumour and reactions in the rest of the body. $\beta$-lactamase enzymes have excellent potential owing to their favourable kinetics and broad substrate specificities, and their absence in mammalian species (Svensson *et al.*, 1992). Another potential advantage is their ability to be competitively inhibited. Svensson *et al.* (1992) used a cephalosporin mustard, which is at least 50 times less cytotoxic than its toxic form, phenylenediamine mustard.

For all the work done in this area of antibody-directed enzyme-prodrug therapy, comparatively little is known on the biodistribution of the various components. Smith and Thijssen (1986) have developed a preliminary mathematical (compartmental) model for the distribution of drugs following local activation. The first and most comprehensive experimental study on the biodistribution of active drugs after site-specific activation of prodrugs is by Antoniw *et al.* (1990). They present data on the concentration of prodrug and drug, and enzyme activity, in nude mice bearing human CC3 choriocarcinoma xenografts, for plasma, tumour, liver, kidney and lung. Their system was A5B7F(ab')$_2$ antibody fragment conjugated to carboxypeptidase G$_2$, which converts a derivative of benzoic acid mustard to its active form. As the importance of *in vivo* transport issues has been acknowledged, more studies measuring the local concentrations of ECA and/or prodrug and drug have been published. Bosslet *et al.* (1992) developed a fusion protein for prodrug activation consisting of a humanised anti-CEA region and human $\beta$-glucuronidase using recombinant DNA technology and have measured the plasma pharmacokinetics and average concentrations in the liver, lung, kidney, intestine, heart and several tumour lines in nude mice (Bosslet *et al.*, 1994). In their *in vivo* study, a long time delay of 7 days between protein and prodrug injection was used to increase specificity as suggested in Yuan *et al.* (1991). Wallace *et al.* (1994) carried out studies in nude mice to determine the *in vivo* conversion of a 5-fluorouracil (5-FU) prodrug 5-fluorocytosine (5-FC), measuring the antibody (L6), prodrug and drug concentrations in the plasma, liver, kidney, lung, spleen, muscle, and in H2981 (L6-binding) and H3719 (L6-non-binding) tumours. In the present study we have compared our model simulations when possible with these diverse sets of experimental data, which include sufficient biodistribution data (Antoniw *et al.*, 1990; Bosslet *et al.*, 1994; Wallace *et al.*, 1994). A key aim of developing this model was to provide a basis for the optimisation of ECA-prodrug therapy. The ability of models to provide novel insight for this approch is evidenced by this quote from Hellström and Senter (1991)

'Yet another variable concerns the kinetics of drug release by the targeted enzyme. It would be of interest to test the validity of recent pharmacokinetic model studies suggesting that low turnover rates may result in higher tumour to blood drug ratios if the conjugate concentration in the tumour is higher than in the blood (Yuan *et al.*, 1991). These same analyses also suggest that under some conditions there may be advantages in enzymes with high $K_m$ values.'

We hope that the present model furthers understanding of the ECA approach.

### Materials and methods

### *Model development*

The tissue is modelled as a Krogh cylinder (Krogh, 1922), in which a microvessel of radius $\rho$ is surrounded by tissue of radius $L$. Diffusion reaction equations are written to describe the extravascular concentrations as a function of both time and radial position. These differential equations (in both tissue and plasma) were solved simultaneously using a numerical finite difference method. The antibody-antigen binding is assumed to be specific, linear and saturable, and the prodrug conversion is assumed to follow Michaelis-Menten kinetics.

There are four extravascular species undergoing unsteady-state diffusion and/or reaction:

$$\frac{\delta C_A}{\delta t} = D_A \nabla^2 C_A - k_f C_A A_g + k_r C_B \quad (1)$$

$$\frac{\delta C_P}{\delta t} = D_P \nabla^2 C_P - \frac{V_{max} C_A C_P}{K_M + C_P} - k_{ns} C_P \quad (2)$$

$$\frac{\delta C_D}{\delta t} = D_D \nabla^2 C_D + \frac{V_{max} C_A C_P}{K_M + C_P} + k_{ns} C_P \quad (3)$$

$$\frac{\delta C_B}{\delta t} = k_f C_A A_g - k_r C_B - k_e C_B \quad (4)$$

where $\nabla^2$ is the Laplacian operator

$$\nabla^2 C \text{ equals} \frac{1}{r}\frac{\delta}{\delta r}\left(r\frac{\delta C}{\delta r}\right)$$

in cylindrical coordinates, $D$ is the effective diffusion coefficient, $t$ is the time after injection, $r$ is the distance from the centre of the blood vessel (which has a radius of $\rho$), $C_A$ is the free antibody concentration, $C_B$ is the bound antibody concentration, $C_P$ is the prodrug concentration, $C_D$ is the drug concentration and $A_g$ is the number of free binding sites on the tumour cells ($A_g = A_g{}^0 - C_B$). Metabolism (antigen internalisation) is treated as a first-order process ($k_e$), that eliminates BFA bound to the tumour-associated antigen; $V_{max}$ and $K_M$ are the Michaelis-Menten rate constants characterising the prodrug conversion reaction in the presence of the ECA enzyme; $k_f$ and $k_r$ are the association and dissociation reaction rate constants for the binding of the ECA to the TAA; and $k_{ns}$ is the rate constant for non-specific conversion of prodrug to drug.

There is a no-flux boundary condition for mobile species at the surface of the cylinder ($r = L$) owing to symmetry with surrounding identical Krogh cylinders:

$$\frac{\delta C_i}{\delta r}\Big|_{r=L} = 0 \quad (i = A, P, D) \tag{5}$$

The boundary condition at the blood vessel wall relates the solute flux to the concentration gradient between the plasma ($C_{i1}$) and interstitial concentrations and the permeability ($P_i$) and partition ($\gamma_i$) coefficients (where $\gamma_i$ is the ratio of the interstitial to plasma concentrations at equilibrium):

$$-D_i \frac{\delta C_i}{\delta r}\Big|_{r=p} = P_i(C_{i1} - C_i|_{r=p}/\gamma_i) \quad (i = A, P, D) \tag{6}$$

Equations must also be developed for the plasma concentrations. Following Baxter *et al.* (1992) we use a three-compartment model with saturable elimination to describe the pharmacokinetics of the low molecular weight agents and a two-compartment model for ECA, allowing for conversion of prodrug to drug in all compartments where enzyme is present:

$$\frac{dC_{i1}}{dt} = -\sum_{j=2}^{3}\{K_{1j,i}(C_{i1} - C_{ij})\} - \phi_i \frac{V_{max} C_{A1} C_{P1}}{K_M + C_{P1}} - K_{10,i} C_{i1} \quad (i = A, P, D) \tag{7}$$

$$\frac{dC_{ij}}{dt} = -K_{j1,i}(C_{ij} - C_{i1}) - \phi_i \frac{V_{max} C_{Aj} C_{Pj}}{K_M + C_{Pj}} - \quad (i = A, P, D; j = 2, 3) \tag{8}$$

where $\phi_A = 0$, $\phi_P = 1$ and $\phi_D = -1$, $j$ represents the three compartments, with $K_{jk,i}$ representing the transfer coefficient of species $i$ from compartment $j$ to $k$ and $K_{10}$ is the rate constant for elimination from the plasma (e.g. via urine). These compartmental transfer coefficients may be obtained from the coefficients of the following expression that provides the best fit to experimental data for the plasma concentration ($C_{i1}$) of free species (Gibaldi and Perrier, 1982). For the antibody, a biexponential fit was sufficient to describe the plasma pharmacokinetics. Without further physiological information on ECA distribution, and with the smaller prodrug able to move between the shallow and deep compartments, it was assumed that there was no ECA in the deep compartment, and hence no prodrug conversion. (Additional simulations without this assumption resulted in no significant change in drug concentration in the tumour or plasma.)

$$\frac{C_{i1}}{C_{i1}|_{t=0}} = \alpha_1 \exp^{-\lambda_1 t} + \alpha_2 \exp^{-\lambda_2 t} + (1 - \alpha_1 - \alpha_2) \exp^{-\lambda_3 t} \quad (i = A, P, D) \tag{9}$$

The initial conditions are zero concentrations in the plasma and tissues for all species, with a step change in the plasma antibody concentration at $t = 0^+$ to $Cp^0$ = dose/plasma volume. Similarly when the prodrug is introduced there is a step change at $t = \tau^+$ from 0 to its initial plasma concentration.

In the model we have not attempted to incorporate pharmacodynamic effects, cellular internalisation or metabolism. These effects, while important, vary tremendously from one prodrug/drug system to another (e.g. depending upon hydrophilicity, membrane transport, charge, mode of action etc.) The model equations could be modified to incorporate these effects for a specific drug but the equations above are meant to be general for a system in which the concentration at the site of action is proportional to the interstitial concentration. If, for example, the drug were taken up in an irreversible fashion by the cells, equation 3 would represent the interstitial drug concentration, with an extra term, $-k_{intern} \times C_{D\text{-}int}$, and another equation for the cellular drug concentration:

$$\frac{\delta C_{D\text{-cell}}}{\delta t} = +k_{intern} \times C_{D\text{-int}} - k_{metab} \times C_{D\text{-cell}}$$

*Model parameters and simulations*

The actual parameters used in the model depend upon the exact enzyme, antibody, prodrug, tumour line etc. Since a main purpose of this study is to compare the microscopic distribution of ECA, drug and prodrug with the distribution in other approaches such as bifunctional antibodies and haptens, we have chosen the baseline model parameters to be as close as possible to those used previously in Baxter *et al.* (1992) and Yuan *et al.* (1991 for enzyme kinetic parameters). These baseline parameters are summarised in Table I, and represent a prodrug with a molecular weight of 600 and an ECA or protein with characteristics similar to an $F(ab')_2$ molecule. Further details on the source of these parameters may be found in Baxter *et al.* (1992) and Yuan *et al.* (1991). The simulations were based on a bolus injection of ECA 72 h before prodrug administration. The equations given above were solved numerically using the IMSL subroutine DMOLCH, which uses the method of lines with cubic hermite polynomials to solve a system of ordinary differential equations obtained from a centered finite difference approximation of the partial differential equations for diffusion (IMSL, 1987).

In order to determine the effects of relevant physiological parameters on the uptake and distribution of ECA and drug we have carried out additional simulations varying each of the important parameters. To allow comparison of different conditions, each profile is analysed to calculate the average concentration ($\langle C \rangle$), area under the concentration–time curve (AUC) for plasma and tumour. Since the ECA-prodrug system may be beneficial only for therapy and not detection, the instantaneous tumour–plasma level (specificity ratio) is not as important as the relative total exposure to the drug, i.e. the therapeutic index ($TI = AUC_{tumour}/AUC_{plasma}$). As in Baxter *et al.* (1992) we have calculated an index of spatial uniformity (UNI), which is equal to the volume around the blood vessel in which one-half of the ECA or drug has distributed, divided by the equivalent volume under a uniform distribution. Thus UNI, a function of time, approaches zero when all the material is just outside of the blood vessel and approaches unity for a uniform interstitial distribution. The role of cellular uptake or elimination of antibody may vary greatly from system to system. To study the effect of antigen internalisation and/or antibody metabolism on the resulting drug distribution in the ECA system we have included a simulation with $k_e = 1.9$ day$^{-1}$ (as used in Baxter and Jain, 1991*a*; Baxter *et al.*, 1992).

### Results

*Baseline simulations*

Figure 2 shows the model simulations for the baseline parameters for the prodrug and drug distribution in which the prodrug is injected 3 days after ECA administration. The spatial distribution is plotted for a series of time points. Note that both the prodrug and drug concentration profiles are quite uniform even at early time points. This is in contrast with the profiles in Figure 3, which show the ECA concentration as well as the hapten concentration following BFA administration with the model from (Baxter *et al.*, 1992). In this figure, the antibody concentrations are essentially constant over the short time that the drug is present. The hapten molecules are mostly bound to the antibody (for low hapten doses, and after the rapid clearance phase for higher doses). Thus the perivascular hapten distribution tends to mimic the antibody distribution. It is also seen that the prodrug and drug molecules clear from the body much more rapidly as they do not bind to the ECA (as in the BFA–hapten approach). The main reason for the very uniform profiles is that the conversion rate >> diffusion rate >> permeability rate for molecules of this size in the presence of a specific enzyme. Figure 4 shows the plasma and tumour concentrations for both drug and prodrug as a function of time. The drug levels are highest in the tumour, where the conversion rates are greatest; hence the prodrug levels in the tumour are less than in the plasma. It can be seen that significant (and detrimental) conversion occurs outside of the tumour for the baseline parameters.

### *Sensitivity analysis*

Other simulations were carried out to determine the effect of various parameters on the drug biodistribution: changes in permeability, diffusivity, binding affinity, kinetic rate constants and the time delay between ECA and prodrug injections. The effect of clearing all antibody from outside the tumour (via plasmapheresis or a chasing antibody) was also studied. The concentration profiles for the prodrug and drug were analysed as described above to yield information on the tumour to plasma concentration ratios (T/P), average concentrations $<C>$, therapeutic index (TI) and perivascular uniformity index (UNI). The results are given in Table II in three sections. First the effect of doses and other clinical parameters was studied. The clearing of ECA from tumour and normal tissues before prodrug administration increased the TI greatly, with only a small decrease in $<C>$. Decreasing the initial dose of ECA was found to increase the TI by reducing prodrug conversion in the plasma. Similarly, waiting 7 days instead of 3 days between ECA and prodrug injections resulted in a much higher TI. Altering the dose of prodrug or drug had marginal effects on the TI, with roughly proportional changes in the $<C>$. An antibody of higher affinity (or with greater antigen expression in the tumour) led to greater ECA concentrations in the tumour and somewhat higher TI. When IgG was used instead of $F(ab')_2$ as the ECA, the TI dropped. This was owing to an increase in the plasma ECA concentration ($6.0 \times 10^{-9}$M *vs* $2.5 \times 10^{-9}$M after 3 days). Next, the enzyme properties were studied. Using enzymes with a lower $V_{max}$ resulted in a better TI, again by minimising conversion of prodrug in the plasma and locations with low antibody concentrations. As less drug would be produced, a greater prodrug dose would be needed to compensate. Clearly, for a very low $V_{max}$ insufficient drug will be produced and the treatment will not be effective. Given a maximum allowable prodrug concentration or AUC and a minimum effective drug concentration or AUC, the model can be used to predict an optimum $V_{max}$ for the enzyme. Finally, physiological parameters were varied to see which might increase the TI. Somewhat surprisingly, decreasing the prodrug and drug permeabilities in the tumour led to a greater TI. This is owing to the longer retention (slower escape) of the drug in the tumour tissue. It is not clear whether lower permeabilities would be more effective in practice, since one cannot always make changes that affect just a single parameter. For example, a drug with a lower permeability would often have a longer plasma clearance half-life. Moderate metabolism (degradation) of the ECA had little effect for a time delay of 3 days but would further reduce the ECA concentration at later times.

These simulations show that the parameters with the greatest effect on the therapeutic index are the $V_{max}$ enzyme rate constant, the time interval before prodrug injection and the reduction in the plasma ECA concentration. All of these parameters are reflections of the requirement to minimise prodrug conversion outside of the tumour, rather than obtain the highest possible conversion or concentration within the tumour. Parameters that affect the therapeutic index to a lesser degree are ECA molecular weight, binding affinity and antibody dose (Table II). Along these lines, Eccles *et al.* (1994) found sustained dose-dependent tumour stasis or regression by waiting 12–14 days between injection of an $IgG_2$-carboxypeptidase ECA and a benzoyl-glutamic acid prodrug in nude mice bearing c-*erb*B-2 breast carcinoma xenografts.

### Discussion

### *Comparison of model with experiments*

Since there are no data available on the perivascular distribution of prodrug and drug following ECA administration, we sought to compare the average concentrations predicted by the model with whole tumour measurements in the literature. There are three sets of published data from

**Table I** Baseline model parameters

| | | *ECA* | | *Prodrug, drug* | |
|---|---|---|---|---|---|
| $D$ (cm$^2$ s$^{-1}$) | Diffusion coefficient | $2.0 \times 10^{-8}$ | Gerlowski and Jain (1986) | $4.3 \times 10^{-6}$ | Nugent and Jain (1984) |
| $P$ (cm s$^{-1}$) | Permeability coefficient | $9.0 \times 10^{-7}$ | Gerlowski and Jain (1986) | $1.0 \times 10^{-4}$ | Jain (1987)[b] |
| $\gamma$ | Partition coefficent | 1.0 | – | 1.0 | – |
| $C_i^0$ (M) | Initial plasma concentration | $2.0 \times 10^{-8}$ | Le Doussal *et al.* (1990) | $1.0 \times 10^{-4}$ | Yuan *et al.* (1991) |
| $\alpha_1$ | Pharmacokinetic parameters for free species: | 0.76 | Stickney *et al.* (1991) | 0.69 | Houston *et al.* (1979) |
| $\alpha_2$ | | 0.24 | | 0.24 | |
| $\lambda_1$ (min$^{-1}$) | | $4.44 \times 10^{-3}$ | (ZCE-025/ CHA-255) | 0.588 | ([$^{113}$In] DTPA) |
| $\lambda_2$ (min$^{-1}$) | $\frac{C_i}{C_i^0} = \alpha_1 \exp(-\lambda_1 t) + \alpha_2 \exp(-\lambda_2 t) +$ | $4.10 \times 10^{-4}$ | | $3.34 \times 10^{-2}$ | |
| $\lambda_3$ (min$^{-1}$) | $(1 - \alpha_1 - \alpha_2)\exp(-\lambda_3 t)$ | – | | $5.20 \times 10^{-3}$ | |
| $\rho$ ($\mu$m) | Blood vessel radius | 10 | | Baxter and Jain (1991*b*); Gerlowski and Jain (1986) | |
| $L$ ($\mu$m) | Intercapillary half-distance | 100 | | Baxter and Jain (1991*b*); Gerlowski and Jain (1986) | |
| $k_f$ (M$^{-1}$ s$^{-1}$) | Association rate constant | $4.33 \times 10^4$ | | Baxter and Jain (1991*a*) | |
| $k_r$ (s$^{-1}$) | Dissociation rate constant | $2.07 \times 10^{-5}$ | | $= k_f/K_a$ | |
| $K_a$ (M$^{-1}$) | Binding affinity | $2.1 \times 10^9$ | | Le Doussal *et al.* (1990) | |
| $k_e$ (s$^{-1}$) | Metabolism/internalisation rate constant | 0 ($2.17 \times 10^{-5}$)[a] | | See text | |
| $A_g^0$ (M) | Antigen density | $1.6 \times 10^{-7}$ | | Le Doussal *et al.* (1990)[b] | |
| $V_{max}$ (min$^{-1}$) | Maximum enzyme conversion rate (velocity) | 6000 | | Senter (1989); Yuan *et al.* (1991) | |
| $K_M$ (M) | Michaelis constant | $1.0 \times 10^{-4}$ | | Nielsen and Bundgaard (1988); Yuan *et al.* (1991) | |
| $\tau$ (h) | Time delay between ECA and prodrug injections | 72 | | Yuan *et al.* (1991) | |

[a]Baseline value is zero, but the simulation which includes metabolism uses a value of $2.17 \times 10^{-5}$ s$^{-1}$ (Baxter and Jain, 1991*a*).
[b]As determined indirectly in Baxter *et al.* (1992).

**Figure 2** Baseline parameter simulations. Prodrug (**a**); drug extravascular concentrations in the tumour (**b**). Theoretical concentrations are given at different times for prodrug injected 3 days after i.v. administration of ECA. Quite uniform concentrations are seen, even at early times after prodrug administration. Model parameters are given in Table I.

**Figure 3** (**a**) The ECA concentration is shown for the same time scales as in Figure 2. (**b**) The concentration of hapten (bound + free) from Baxter *et al.* (1992) is shown for comparison with the ECA approach. The relevant time scales are much longer for the BFA–hapten system.

different groups that contain sufficient information (data and parameters) to compare with our model (Antoniw *et al.*, 1990; Bosslet *et al.*, 1994; Wallace *et al.*, 1994). These three studies look at different ECA and prodrug systems in nude mice. All have taken some measures to address the problem of non-specific conversion of prodrug outside the tumour. Antoniw *et al.* (1990) and Bosslet *et al.* (1994) use long time intervals before prodrug administration (6 and 7 days respectively), whereas Wallace *et al.* (1994) use an anti-idiotypic antibody to clear the ECA from the bloodstream. Bosslet *et al.* (1994) also used an enzyme with a relatively low $V_{max}$. In the simulations described below, baseline parameters were used unless different parameters were given in the experimental study.

*Carboxypeptidase $G_2$–benzoic acid mustard* Antoniw *et al.* (1990) were the first to present the pharmacokinetics of ECA, prodrug and drug together. They used carboxypeptidase $G_2$ conjugated to $F(ab')_2$–anti-human chorionic gonadotropin antibody W14A to convert the prodrug 4-(bis (2-chloroethyl) amino) benzoyl-L-glutamic acid to its activated derivative, benzoic acid mustard. The prodrug was administered (41 µmol $kg^{-1}$ i.p.) to mice bearing human CC3 choriocarcinoma xenografts (0.5–1.0 g in size) 6 days after i.v. injection of 1000 U $kg^{-1}$ ECA. The baseline parameters were used with the following changes in the enzyme kinetics and small molecule plasma pharmacokinetics: $V_{max} = 0.62 \times 10^6$ µmol $U^{-1}$ $min^{-1}$; $K_M = 4.5$ µM (Springer *et al.*, 1991); prodrug $\alpha_1 = 0.9716$, $\alpha_2 = 0.0162$, $\lambda_1 = 0.5$ $min^{-1}$, $\lambda_2 = 0.0334$ $min^{-1}$, $\lambda_3 = 0.0052$ $min^{-1}$; drug $\alpha_1 = 0.9716$, $\alpha_2 = 0.0162$, $\lambda_1 = 0.5$ $min^{-1}$, $\lambda_2 = 0.0334$ $min^{-1}$, $\lambda_3 = 0.0052$ $min^{-1}$ (fitted from pharmacokinetic data). $V_{max}$ was converted to units of $min^{-1}$ by assuming an initial enzyme plasma concentration of $2.0 \times 10^{-8}$. (This choice is arbitrary but has no effect on the results as long as the dose is below that needed to saturate the tumour-associated antigen.) The model simulation for these parameters is compared with the data in Figure 5, for a drug partition coefficient of 0.17 [data values with standard errors from Antoniw *et al.* (1990), kindly provided by Dr C Springer]. The partition coefficient was estimated by fitting the model to the data; this value reflects at least four physiological or physicochemical factors. First, at equilibrium the drug concentrations in the vascular and extravascular spaces need not be the same; the ratio of the interstitial to plasma equilibrium concentration is defined as the partition coefficient, $\gamma_i$. Second, the uptake of prodrug may well be flow limited in the tumour, i.e. the uptake may be affected not by the permeability but by the regional blood flow rates (Yuan *et al.*, 1993). Third, the tumour may have necrotic or poorly perfused regions that have little or no blood supply and hence reduced availability of prodrug. Fourth, the extent to which the prodrug and drug are internalised by cells will have an effect on the measured concentration, which is an average of vascular, intersitial and cellular fractions. Hence, the estimated $\gamma_i$ is not necessarily the same as the true equilibrium partition coefficient.

**Figure 4** Plasma concentrations of prodrug and drug are plotted as a function of time, 3 days after injection of ECA. The same baseline parameters were used (Table I) as in previous figures.

**Figure 5** Comparison with CG2-BA system of Antoniw *et al.* (1990). The theoretical plasma (dotted line) and tumour extravascular (dashed line) concentrations were calculated using $\gamma_{drug} = 0.17$ (see text for other parameters). The experimental drug tissue concentrations in mice bearing human CC3 choriocarcinoma xenografts for plasma (■) and tumour (○) are given up to 12 h after prodrug administration.

**Table II** Analytical indices from simulations[a]

| Case | T/P Prodrug | T/P Drug | <C> ($10^{-5}$ M) Prodrug | <C> ($10^{-5}$ M) Drug | UNI Prodrug | UNI Drug | AUC[b] ($10^{-5}$ M min) Prodrug | AUC[b] ($10^{-5}$ M min) Drug | TI[b] Prodrug | TI[b] Drug |
|---|---|---|---|---|---|---|---|---|---|---|
| *Δ Doses* | | | | | | | | | | |
| Baseline | 0.138 | 1.23 | 0.0035 | 1.79 | 0.994 | 1.00 | 4.36 | 205 | 0.12 | 1.14 |
| $C_{p}{}^{0}{}_{A} \div 100$ | 1.16 | 2.34 | 1.64 | 0.157 | 1.00 | 1.00 | 182 | 28.3 | 0.92 | 1.54 |
| $C_{p}{}^{0}{}_{P} \times 10$ | 0.162 | 1.27 | 0.129 | 17.9 | 0.994 | 1.00 | 94.4 | 2000 | 0.17 | 1.24 |
| $C_{p}{}^{0}{}_{P} \div 10$ | 0.136 | 1.23 | 0.0003 | 0.18 | 0.994 | 1.00 | 0.38 | 20.6 | 0.12 | 1.12 |
| $C_{p}{}^{0}{}_{A} \times 10$ and $C_{p}{}^{0}{}_{P} \times 10$ | 0.0136 | 1.22 | 0.0003 | 18.0 | 0.88 | 1.00 | 2.41 | 2090 | 0.012 | 1.06 |
| $K_a \times 10$ | 0.14 | 1.60 | 0.055 | 1.74 | 0.998 | 1.00 | 9.12 | 200 | 0.12 | 1.41 |
| IgG (instead of Fab'$_2$)[c] | 0.067 | 1.22 | 0.0006 | 1.80 | 0.988 | 1.00 | 1.65 | 208 | 0.068 | 1.08 |
| 1 week between ECA and prodrug | 0.128 | 11.6 | 0.17 | 1.63 | 0.994 | 1.00 | 22.1 | 187 | 0.12 | 5.3 |
| Clear ECA outside tumour | 0.137 | ∞ | 0.202 | 1.60 | 0.993 | 1.00 | 28.2 | 181 | 0.13 | ∞ |
| *Δ Enzyme properties* | | | | | | | | | | |
| $V_{max} \times 100$ | 0.0009 | 0.45 | 1.4e-8 | 1.79 | 0.37 | 1.00 | 0.0014 | 213 | 0.009 | 0.94 |
| $K_M \div 100$ | 0.0009 | 1.22 | 1.9e-7 | 1.79 | 0.37 | 1.00 | 0.021 | 210 | 0.0012 | 1.06 |
| Baseline | 0.138 | 1.23 | 0.0035 | 1.79 | 0.994 | 1.00 | 4.36 | 205 | 0.12 | 1.14 |
| $V_{max} \div 100$, $K_M \div 100$ | 0.83 | 2.43 | 0.93 | 0.87 | 1.00 | 1.00 | 62.2 | 148 | 0.56 | 1.41 |
| $V_{max} \div 100$ | 1.16 | 2.34 | 1.64 | 0.157 | 1.00 | 1.00 | 182 | 28.3 | 0.92 | 1.54 |
| $V_{max} \div 10\,000$ | 1.21 | 2.40 | 1.80 | 0.0016 | 1.00 | 1.00 | 210 | 0.32 | 0.97 | 1.55 |
| *Δ Physiological parameters* | | | | | | | | | | |
| Baseline | 0.138 | 1.23 | 0.0035 | 1.79 | 0.994 | 1.00 | 4.36 | 205 | 0.12 | 1.14 |
| $D_P$ and $D_D \times 100$ | 0.138 | 1.23 | 0.0035 | 1.79 | 0.994 | 1.00 | 4.36 | 205 | 0.12 | 1.14 |
| $D_P$ and $D_D \div 100$ | 0.032 | 0.86 | 0.0008 | 1.25 | 0.385 | 0.992 | 1.10 | 196 | 0.030 | 1.09 |
| $P_P$ and $P_D \times 100$ | 0.750 | 1.02 | 0.0190 | 1.48 | 0.994 | 1.00 | 28.2 | 187 | 0.78 | 1.04 |
| $P_P$ and $P_D \div 100$ | 0.016 | 0.51 | 0.0004 | 0.75 | 0.994 | 1.00 | 0.50 | 219 | 0.014 | 1.22 |
| $A_g{}^0 \times 10$ | 0.139 | 1.60 | 0.0550 | 1.74 | 0.999 | 1.00 | 9.05 | 200 | 0.122 | 1.41 |
| Metabolism ($k_e = 1.87$ day$^{-1}$) | 1.21 | 1.22 | 0.48 | 1.32 | 1.00 | 1.00 | 50.9 | 159 | 0.69 | 1.12 |
| *Comparison with experimental results* | | | | | | | | | | |
| Baseline | 0.138 | 1.23 | 0.0035 | 1.79 | 0.994 | 1.00 | 36.4 | 180 | 0.12 | 1.14 |
| Simulation of Antoniw *et al.* (1990) | 5.4e$^{-5}$ | 0.176 | 4.2e-7 | 0.667 | 0.18 | 1.00 | 0.012 | 120 | 7.3e-5 | 0.21 |
| Simulation of Wallace *et al.* (1994) | 0.041 | 71.1 | 64.6 | 606 | 0.998 | 1.00 | 5900 | 40000 | 0.025 | 2.18 |
| Simulation of Bosslet *et al.* (1992) | 0.15 | 4.47 | 7.88 | 0.464 | 1.00 | 1.00 | 1770 | 390 | 0.22 | 11.3 |

[a]Indices shown are spatial averages, for time = 30 min post prodrug injection (which was 72 h after ECA injection), except where otherwise noted. Tumour–plasma concentration ratio, average concentration, spatial uniformity index, area under the curve and therapeutic index are reported. The therapeutic index of the drug (ratio of AUC in tumour *vs* AUC in plasma) is an important overall measure of the effectiveness/specificity of an ECA–prodrug therapy. [b]AUC and TI based on exponential extrapolation after first 3 h, to estimate the total exposure. [c]Model parameters for IgG were similar to baseline, with the following changes: double $K_a$, one-half $A_g{}^0$, $\alpha_1 = 0.46$, $\lambda_1 = 0.0117$ min$^{-1}$, $\lambda_2 = 0.000135$ min$^{-1}$ (for B72.3) (Shockley *et al.*, 1992); D = $1.39 \times 10^{-8}$ cm$^2$ s$^{-1}$ (Clauss and Jain, 1990); P = $5.73 \times 10^{-7}$ cm s$^{-1}$ (Baxter and Jain, 1989; Gerlowski and Jain, 1986).

*Glucuronidase–fusion protein – doxorubicin* Bosslet *et al.* (1994) developed a novel fusion protein with a humanised carcinoembryonic antigen-specific variable region and the enzymatic activity similar to that of human $\beta$-glucuronidase. This ECA was used to convert a non-toxic glucuronide-spacer derivative of doxorubicin. The prodrug was injected (i.v. 250 mg $kg^{-1}$) 7 days after the i.v. injection of fusion protein (20 mg $kg^{-1}$). The baseline model parameters were used with the exception of a lower $V_{max}=0.635$ nmol $min^{-1}\times\mu g$ (converted to 159 $min^{-1}$); $K_M=1.3$ mM (Bosslet *et al.*, 1994); $C_p^0=1.44\times10^{-6}$ M; ECA $\alpha_1=0.68$, $\alpha_2=0.32$, $\lambda_1=0.0283$ $min^{-1}$, $\lambda_2=0.00161$ $min^{-1}$; prodrug $\alpha_1=0.99956$, $\alpha_2=0.00044$, $\lambda_1=0.076$ $min^{-1}$, $\lambda_2=0.00064$ $min^{-1}$; drug $\alpha_1=0.999$, $\alpha_2=0.0001$, $\lambda_1=0.48$ $min^{-1}$, $\lambda_2=0.00167$ $min^{-1}$ (fit from pharmacokinetic data). In addition, a lower antibody-binding affinity of $1.0\times10^8$ $M^{-1}$ and an antigen concentration of $3.0\times10^{-8}$ M were used to better fit the *in vivo* ECA concentration profiles, with a permeability coefficient of $3\times10^{-7}$ cm $s^{-1}$ (3-fold lower than baseline) and a partition coefficient fit as 0.17 (Figure 6a). Bosslet *et al.* (1992) measured $K_a$ *in vitro* to be $1.0\times10^{10}$ $M^{-1}$ for the fusion protein, which is used in the model predicts a much higher concentration 7 days after injection. Figure 6b shows the prodrug and drug concentrations compared with the model simulations. An 80-fold lower permeability coefficient and a partition coefficient of 0.33 were required for the prodrug, and a 20-fold higher $V_{max}$ (3180 $min^{-1}$), in order to match the experimental data. The drug permeability was unchanged and its partition coefficient was 1.0. The reduced apparent prodrug permeability is indicative of a high degree of plasma protein binding, and/or a strongly flow-limited uptake. The effect of these adjusted parameters may be summarised as follows: the low $A_g^0$ and permeability are implied by the low tumour ECA concentrations at early time points (lest the model concentrations of the bound or free ECA become higher than measured); the low $K_a$ and low partition coefficient are implied by the decreased tumour concentration at later time points (if $K_a$ or $\gamma$ were higher, the bound and free ECA concentrations respectively, would be too high at 7 days after injection); the low prodrug permeability is required owing to the low tumour prodrug concentrations at 30–60 min; the low prodrug partition coefficient serves to reduce the prodrug concentration at 4 and 8 h; and the higher prodrug conversion rate ($V_{max}$) is needed to produce the measured tumour drug concentrations for these ECA and prodrug concentrations.

**Figure 6** Comparison with Glu-Dox system of Bosslet *et al.* (1994). (**a**) ECA concentration in the plasma (model, dot-dash lime; data, ◆) tumour (model, solid line; data, ○) up to 7 days after i.v. injection of 20 mg $kg^{-1}$ fusion protein. (**b**) Prodrug: (plasma model, solid line; plasma data, ●; tumour model, dashed line; tumour data, ▷) and drug (tumour model, solid line; tumour data, □) concentrations up to 8 hours after i.v. injection of 250 mg $kg^{-1}$ prodrug.

*Cytosine deaminase–L6 – 5-fluorouracil* Wallace *et al.* (1994) used an anti-idiotypic antibody that could bind to, and help remove, circulating ECA for a cytosine deaminase enzyme–L6 monoclonal antibody conjugate for converting 5-FC to 5-FU. Nude mice bearing H2981 lung adenocarcinoma were used. The use of the clearing antibody (13B) to remove ECA from outside the tumour gave excellent tumour–plasma ratios for 5-FU. The baseline model parameters were used with the exception of $V_{max}=3000$ $min^{-1}$ and $K_M=2.5$ mM [arbitrary estimates, values not given by Wallace *et al.* (1994)]; the same pharmacokinetic parameters and reduced small molecule permeability were used as above in the fusion protein simulations. The use of a third step, the clearing of the ECA from the blood, would be very difficult to determine without a good whole body pharmacokinetic model. Therefore to allow comparision we used the experimental value of ECA plasma concentration 24 h after injection of 200 $\mu$g (i.p.) 13B, which was 24 h after injection of

**Figure 7** Comparison with CD-L6–5FU system of Wallace *et al.* (1994). Prodrug (plasma model, dashed line; plasma data, *; tumour model, dashed line; tumour data, ⊠) and drug (plasma model, dot-dash line; plasma data, ○; tumour model, dotted line; tumour data, ■) concentrations up to 90 min after i.v. injection of 800 mg $kg^{-1}$ 5-FC prodrug.

300 μg (i.v.) L6-CD ECA, and assumed that the concentration in normal tissues was reduced by an equivalent amount (approximtely 41 times lower). Figure 7 shows a comparison between the simulations and the data. The prodrug concentrations were given only at one time point (30 min) in the two-step approach. A partition coefficient of 0.17 for the drug was found to give a good agreement between theory and experiment for the drug plasma concentration.

*Summary*

The present model and simulations have served two purposes: (i) a comparison of the perivascular distribution of active agents for the BFA and ECA two-step approaches shows much more uniform drug concentrations in the ECA approach, with higher absolute concentration levels achievable; (ii) a comparison of the model results averaged spatially with data for a variety of experimental systems highlights the importance of minimising the ECA concentration external to the tumour. In particular the effective use of a clearing antibody, as in Wallace *et al.* (1994) gives a dramatically higher tumour–plasma ratio and therapeutic index. In practice, residual amounts of ECA in normal tissues that may escape a clearing antibody and convert prodrug and/or return to the bloodstream, may be a limiting factor. A strategy combining an enzyme with low $V_{max}$ and a 1 week time delay between ECA and prodrug injections was also seen to produce a good therapeutic ratio (Bosslet *et al.*, 1994), by minimising prodrug conversion outside of the tumour. The ECA approach was found to have a higher and more uniform concentration than the hapten with BFA, with greater specificity as well. One potential disadvantage with the ECA system compared with the use of radionuclides is the difficulty of targeting cells distant from a patent blood vessel; drug-resistant cells may be a problem for some prodrugs, although the use of prodrugs of aklylating agents may alleviate multidrug-resistant cells (Springer *et al.*, 1994). In addition, ECA with prodrug is also not used for cancer detection. Exact quantitative agreement between these simulations and experimental data has been hindered by the assumption of homogeneous transport properties throughout all regions of a tumour and by the simplified plasma pharmacokinetic model (and transcapillary exchange) used, as has been addressed elsewhere (Jain and Baxter, 1988; Baxter and Jain, 1989; Strand *et al.*, 1993; Baxter *et al.*, 1994, 1995). It is hoped that this model will help to improve the effectiveness of cancer therapies based on ECA–prodrug systems.

**Abbreviations**

BA, benzoic acid mustard; BFA, bifunctional antibody; CEA, carcinoembryonic antigen; CG2, carboxypeptidase; Dox, doxorubicin; ECA, enzyme-conjugated antibody; Glu, glucuronidase; MAb, monoclonal antibody; TAA, tumour associated antigen; 5-FC, 5-fluorocytosine; 5-FU, 5-fluorouracil; %i.d. $g^{-1}$, percentage of injected dose per gram in an organ or tissue.

**Acknowledgements**

The authors would like to thank Drs Jim Baish and Fan Yuan for their helpful comments on this manuscript. This work was supported by a grant NCI (R35-CA-56591). This work was presented at the 86th Annual Meeting of the American Institute of Chemical Engineers, San Francisco, CA, 13–18 November 1994.

**References**

ANTONIW P, SPRINGER CJ, BAGSHAW KD, SEARLE F, MELTON RG, ROGERS GT, BURKE PJ AND SHERWOOD RF. (1990). Disposition of the prodrug 4-(bis (2-chloroethyl) amino) benzoyl-L-glutamic acid and its active parent drug in mice. *Br. J. Cancer*, **62**, 909–914.

BAGSHAWE KD. (1987). Antibody directed enzymes revive anti-cancer prodrugs concept. *Br. J. Cancer*, **56**, 531–532.

BAGSHAWE KD, SPRINGER CJ, SEARLE F, ANTONIW P, SHARMA SK, MELTON RG AND SHERWOOD RF. (1988). A cytotoxic agent can be generated selectively at cancer sites. *Br. J. Cancer*, **58**, 700–703.

BAXTER LT AND JAIN RK. (1989). Transport of fluid and macromolecules in tumors. I. Role of interstitial pressure and convection. *Microvasc. Res.*, **37**, 77–104.

BAXTER LT AND JAIN RK. (1990). Transport of fluid and macromolecules in tumors. II. Role of heterogeneous perfusion and lymphatics. *Microvasc. Res.*, **40**, 246–263.

BAXTER LT AND JAIN RK. (1991*a*). Transport of fluid and macromolecules in tumors. III. Role of binding and metabolism. *Microvasc. Res.*, **41**, 5–23.

BAXTER LT AND JAIN RK. (1991*b*). Transport of fluid and macromolecules in tumors. IV. A microscopic model of the perivascular distribution. *Microvasc. Res.*, **41**, 252–272.

BAXTER LT, YUAN F AND JAIN RK. (1992). Pharmacokinetic analysis of the perivascular distribution of bifunctional antibodies and haptens: Comparison with experimental data. *Cancer Res.*, **52**, 5838–5844.

BAXTER LT, ZHU H, MACKENSEN DG AND JAIN RK. (1994). Physiologically based pharmacokinetic model for specific and non-specific monoclonal antibody fragments in normal tissues and human tumor xenografts in nude mice. *Cancer Res.*, **54**, 1517–1528.

BAXTER LT, ZHU H, MACKENSEN DG, BUTLER WF AND JAIN RK. (1995). Biodistribution of monoclonal antibodies: scale-up from mouse to man using a physiologically-based pharmacokinetic model. *Cancer Res.*, **55**, 4611–4622.

BIGNAMI GS, SENTER PD, GROTHAUS PG, FISCHER KJ, HUMPHREYS T AND WALLACE PM. (1992). *N*-(4′-hydroxyphenylacetyl) palytoxin: a palytoxin prodrug that can be activated by a monoclonal antibody-penicillin G amidase conjugate. *Cancer Res.*, **52**, 5759–5764.

BOSSLET K, CZECH J, LORENZ P, SEDLACEK HH, SCHUERMANN M AND SEEMANN G. (1992). Molecular and functional characterization of a fusion protein suited for tumor specific prodrug activation. *Br. J. Cancer*, **65**, 234–238.

BOSSLET K, CZECH J AND HOFFMAN D. (1994). Tumor-selective prodrug activation by fusion protein-mediated catalysis. *Cancer Res.*, **54**, 2151–2159.

CLAUSS MA AND JAIN RK. (1990). Interstitial transport of rabbit and sheep antibodies in normal and neoplastic tissues. *Cancer Res.*, **50**, 3487–3492.

ECCLES SA, COURT WJ, BOX GA, DEAN CJ, MELTON RG AND SPRINGER CJ. (1994). Regression of established breast carcinoma xenografts with antibody-directed enzyme prodrug therapy against c-*erb*B2 p185. *Cancer Res.*, **54**, 5171–5177.

FUJIMORI K, COVELL DG, FLETCHER JE AND WEINSTEIN JN. (1989). Modeling analysis of the global and microscopic distribution of Immunoglobulin G,F(ab′)$_2$, and Fab in tumors. *Cancer Res.*, **49**, 5656–5663.

FUJIMORI K, COVELL D, FLETCHER J AND WEINSTEIN J. (1990). A modeling analysis of monoclonal antibody percolation through tumors: a binding-site barrier. *J. Nucl. Med.*, **31**, 1191–1198.

GERLOWSKI LE AND JAIN RK. (1986). Microvascular permeability of normal and neoplastic tissues. *Microvasc. Res.*, **31**, 288–305.

GIBALDI M AND PERRIER D. (1982). *Pharmacokinetics*, Vol. 15, Drugs and the Pharmaceutical Sciences. Marcel Dekker: New York.

HAISMA HJ, BOVEN E, van MUIJEN M, de JONG J, van der VIJGH WJF AND PINEDO HM. (1992). A monoclonal antibody–β-glucoronidase conjugate as activator of the prodrug epirubicin-glucoronide for specific treatment of cancer. *Br. J. Cancer*, **66**, 474–478.

HELLSTRÖM KE AND SENTER PD. (1991). Activation of prodrugs by targeted enzymes. *Eur. J. Cancer*, **27**, 1342–1343.

HNATOWICH DJ, VIRZI F AND RUSCKOWSKI M. (1987). Investigations of avidin and biotin for imaging applications. *J. Nucl. Med.*, **28**, 1294–1302.

HOUSTON AS, SAMPSON WFD AND MacLEOD MA. (1979). A compartmental model for the distribution of $^{113m}$In-DTPA and $^{99m}$Tc-(Sn)DTPA in man following intraveneous injection. *Int. J. Nucl. Biol.*, **6**, 85–95.

IMSL. (1987). *IMSL Problem-solving Software Systems*, Version 1.0. IMSL: Houston, TX.

JAIN RK. (1987). Transport of molecules across tumor vasculature. *Cancer Metastasis Rev.*, **6,** 559–594.

JAIN RK. (1994). Barriers to drug delivery in solid tumors. *Sci. Am.*, **271,** 58–65.

JAIN RK AND BAXTER LT. (1988). Mechanisms of heterogeneous distribution of monoclonal antibodies and other macromolecules in tumors: significance of elevated interstitial pressure. *Cancer Res.*, **48,** 7022–7032.

KERR DE, SENTER PD, BURNETT WV, HIRSCHBERG DL, HELLSTRÖM I AND HELLSTRÖM KE. (1990). Antibody–penicillin-V-amidase conjugates kill antigen positive tumor cells when combined with doxorubicin phenyoxyacetamide. *Cancer Immunol. Immunother.*, **50,** 6944–6948.

KROGH A. (1922). *The Anatomy and Physiology of Capillaries.* Yale University Press: New Haven, CT.

LE DOUSSAL JM, GRUAZ-GUYON A, MARTIN M, GAUTHEROT E, DELAAGE M AND BARBET J. (1990). Targeting of indium 111-labeled bivalent hapten to human melanoma mediated by bispecific monoclonal antibody conjugates: imaging of tumors hosted in nude mice. *Cancer Res.*, **50,** 3445–3452.

NIELSEN NM AND BUNDGAARD H. (1988). Glycolamide esters as biolabile prodrugs of carboxylic acid agents: synthesis, stability, bioconversion, and physicochemical properties. *J. Pharm. Sci.*, **77,** 285–298.

NUGENT LJ AND JAIN RK. (1984). Extravascular diffusion in normal and neoplastic tissues. *Cancer Res.*, **44,** 238–244.

PAGANELLI G, MAGNANI P, ZITO F, VILLA E, SUDATI F, LOPALCO L, ROSSETTI C, MALCOVATI M, CHIOLERIO F, SECCEMANI E, SICCARDI AG AND FAZIO F. (1991). Three-step monoclonal antibody tumor targeting in carcinoembryonic antigen-positive patients. *Cancer Res.*, **51,** 5960–5966.

SAHIN U, HARTMANN F, SENTER P, POHL C, ENGERT A, DIEHL V AND PFREUNDSCHUH M. (1990). Specific activation of the prodrug mitomycin phosphate by a bispecific anti-CD30/anti-alkaline phosphatase monoclonal antibody. *Cancer Res.*, **50,** 6944–6948.

SANDS H. (1992). Radiolabeled monoclonal antibodies for cancer therapy and diagnosis: is it really a chimera? *J. Nucl. Med.*, **33,** 29–32.

SENTER PD. (1989). Enhancement of the in vitro and in vivo antitumor activities of phosphorylated mitomycin C and etoposide derivatives by monoclonal antibody–alkaline phosphatase conjugates. *Cancer Res.*, **49,** 5789–5792.

SENTER PD, SAULNIER MG, SCHREIBER GJ, HIRSCHBERG DL, BROWN JP, HELLSTROM I AND HELLSTROM KE. (1988). Anti-tumor effects of antibody–alkaline phosphatase conjugates in combination with etoposide phosphate. *Proc. Natl Acad. Sci. USA*, **85,** 4842–4846.

SENTER PD, WALLACE PM, SVENSSON HP, VRUDHULA VM, KERR DE, HELLSTRÖM, I & HELLSTRÖM, KE. (1992). Generation of cytotoxic agents by targeted enzymes. *Bioconj. Chem.*, **4,** 3–9.

SHEPHERD TA, JUNGHEIM LN, MEYER DL AND STARLING JJ. (1991). A novel targeted delivery system utilizing a cephalosporin-oncolytic prodrug activated by an antibody–$\beta$-lactamase conjugate for the treatment of cancer. *Bioorg. Med. Chem. Lett.*, **1,** 21–26.

SHOCKLEY TR, LIN K, SUNG C, NAGY JA, TOMPKINS RG, DEDRICK RL, DVORAK HF AND YARMUSH ML. (1992). A quantitative analysis of tumor specific monoclonal antibody uptake by human melanoma xenografts: effects of antibody immunological properties and tumor antigen expression levels. *Cancer Res.*, **52,** 357–366.

SMITH J AND THIJSSEN H. (1986), Spatial control of drug action: theoretical considerations on the pharmacokinetics of the target-aimed drug. In *Rate-controlled Drug Administration and Action*, Struyker-Boudier, H (ed.) p. 83. CRC Press: Boca Raton, FL.

SPRINGER CJ, BAGSHAWE KD, SHARMA SK, SEARLE F, BODEN JA, ANTONIW P, BURKE PJ, ROGERS GT, SHERWOOD RF AND MELTON RG. (1991). Ablation of human choriocarcinoma xenografts in nude mice antibody-directed enzyme prodrug therapy (ADEPT) with three novel compounds. *Eur. J. Cancer*, **27,** 1361–1366.

SPRINGER CJ, NICULESCU-DUVAZ I AND PEDLEY RB. (1994). Novel prodrugs of alkylating agents derived from 2-fluoro- and 3-fluorobenzoic acids for antibody-directed enzyme prodrug therapy. *J. Med. Chem.*, **37,** 2361–2370.

STICKNEY DR, SLATER JB, KIRK GA, AHLEM C, CHANG CH AND FRINCKE JM. (1989). Bifunctional antibody: ZCE/CHA $^{111}$Indium BLEDTA-IV clinical imaging in colorectal carcinoma. *Antibod. Immunoconj. Radiopharm.*, **2,** 1–13.

STICKNEY DR, ANDERSON LD, SLATER JB, AHLEM CN, KIRK GA, SCHWEIGHARDT SA AND FRINCKE, JM. (1991). Bifunctional antibody: a binary radiopharmaceutical delivery system for imaging colorectal carcinoma. *Cancer Res.*, **51,** 6650–6655.

STRAND S-E, ZANZONICO P AND JOHNSON T. (1993). Pharmacokinetic modeling. *Med. Phys.*, **20,** 515–527.

SUNG C, SHOCKLEY TR, MORRISON PF, DVORAK HF, YARMUSH ML AND DEDRICK RL. (1992). Predicted and observed effects of antibody affinity and antigen density on monoclonal antibody uptake in solid tumors. *Cancer Res.*, **52,** 377–384.

SUNG C, van OSDOL WW, SAGA T, NEUMANN RD, DEDRICK RL AND WEINSTEIN JN. (1994). Streptavidin distribution in metastatic tumors pretargeted with a biotinylated monoclonal antibody: theoretical and experimental phamacokinetics. *Cancer Res.*, **54,** 2166–2175.

SVENSSON HP, KADOW JF, VRUDHULA VM, WALLACE PM AND SENTER PD. (1992). Monoclonal antibody–$\beta$-lactamase conjugates for the activation of a cephalosporin mustard prodrug. *Bioconj. Chem.*, **3,** 176–181.

van OSDOL W, FUJIMORI K AND WEINSTEIN JN. (1991). An analysis of monoclonal antibody distribution in microscopic tumor nodules: consequences of a 'binding site barrier'. *Cancer Res.*, **51,** 4776–4784.

van OSDOL WW, SUNG C, DEDRICK RL AND WEINSTEIN JN. (1993). A distributed pharmacokinetic model of two-step imaging and treatment protocols: application to streptavidin-conjugated monoclonal antibodies. *J. Nucl. Med.*, **34,** 1552–1564.

VRUDHULA VM, SENTER PD, FISCHER KJ AND WALLACE PM. (1993). Prodrugs of doxorubicin and melphalan and their activation by a monoclonal antibody–penicillin-G amidase conjugate. *J. Med. Chem.*, **36,** 919–923.

WALLACE PM AND SENTER PD. (1991). In vitro and in vivo activities of monoclonal antibody–alkaline phosphatase conjugates in combination with phenol mustard phosphate. *Bioconj. Chem.*, **2,** 349–352.

WALLACE PM, MACMASTER JF, SMITH VF, KERR DE, SENTER PD AND COSAND WL. (1994). Intratumoral generation of 5-fluorouracil mediated by an antibody–cytosine deaminase conjugate in combination with 5-fluorocytosine. *Cancer Res.*, **54,** 2719–2723.

WANG S-M, CHERN J-W, YEH M-Y, NG JC, TUNG E AND ROFFLER SR. (1992). Specific activation of glucuronide prodrugs by antibody-targeted enzyme conjugates for cancer therapy. *Cancer Res.*, **52,** 4484–4491.

WEINSTEIN J AND van OSDOL W. (1992). Early intervention in cancer using monoclonal antibodies and other biological ligands: micropharmacology and the 'binding-site barrier'. *Cancer Res.*, **52,** 2747s–2751s.

YUAN F, BAXTER LT AND JAIN RK. (1991). Pharmacokinetic analysis of two-step aproaches using bifunctional and enzyme-conjugated antibodies. *Cancer Res.*, **51,** 3119–3130.

YUAN F, LEUNIG M, BERK D AND JAIN RK. (1993). Microvascular permeability of albumin, vascular surface area and vascular volume measured in human adenocarcinoma LS174T using dorsal chamber in SCID mice. *Microvasc. Res.*, **45,** 269–289.

**Appendix A Nomenclature**

| | |
|---|---|
| $A_g^0$ | $A_g$ ($t=0$) = total (initial) concentration of binding sites in the extravascular space |
| $C_A$ | Interstitial concentration of free (unbound) ECA (M) |
| $C_P$ | Interstitial concentration of prodrug (M) |
| $C_D$ | Interstitial concentration of drug (M) |
| $C_B$ | Interstitial concentration of bound ECA (M) |

| | |
|---|---|
| $C_{ij}$ | Concentration of the free species above [$i$=A (ECA), P (prodrug) or D (drug) (M)] where $j$=1,2,3 represents the plasma, well-perfused and poorly perfused peripheral compartments respectively |
| $<C>$ | Interstitial concentration averaged over the Krogh cylinder (M) |
| $D_i$ | Interstitial diffusion coefficient for the mobile species ($i$=A,P,D) ($cm^2 s^{-1}$) |
| $k_e$ | Elimination (metabolism) rate constant for ECA bound to TAA, $s^{-1}$ |
| $k_f, k_r$ | Forward and reverse binding rate constants for ECA–TAA, $M^{-1} s^{-1}$ and $s^{-1}$ respectively |
| $K_{jk,i}$ | Transfer coefficient of species $i$ from compartment $j$ to $k$ for plasma pharmacokinetics |
| $K_a$ | Binding affinity for ECA–TAA ($=k_f/k_r$) |
| $L$ | One-half of mean intercapillary distance (also radius of Krogh cylinder), ($\mu$m) |
| $P_i$ | Effective vascular permeability coefficient for the mobile species ($i$=A,P,D), ($cm\, s^{-1}$) |
| $r$ | Radial position (distance from centre of blood vessel) ($\mu$m) |
| $S/V$ | Surface area per unit volume for transcapillary exchange ($cm^{-1}$) |
| $t$ | Time (min) |
| $V_{max}, K_M$ | Michaelis–Menten kinetic constants, $min^{-1}$ and $M^{-1}$ respectively. $V_{max}$ here is defined per unit enzyme concentration (and may be converted from other units, e.g. nmol substrate per $\mu$g protein; if given as $\mu$mol substrate per unit enzyme activity, the 'concentration' of enzyme must also be given in terms of unit activity, or an arbitrary proportionality constant used); $K_M$ is also the prodrug concentration at which $V=V_{max}/2$ |
| $\alpha_i \lambda_i$ | Pharmacokinetic constants for free species in plasma ($i$=A,P,D) ($\lambda_i$ in $min^{-1}$) |
| $\gamma_i$ | Partition coefficient between interstitial and vascular space, =1 unless otherwise indicated |
| $\rho$ | Blood vessel radius ($\mu$m) |
| $\tau$ | Time interval between the ECA and prodrug injections, (h) |